# Study and Physical Mapping of the Species-Specific Tandem Repeat CS-237 Linked with 45S Ribosomal DNA Intergenic Spacer in *Cannabis sativa* L.

**DOI:** 10.3390/plants11111396

**Published:** 2022-05-24

**Authors:** Oleg S. Alexandrov, Dmitry V. Romanov, Mikhail G. Divashuk, Olga V. Razumova, Daniil S. Ulyanov, Gennady I. Karlov

**Affiliations:** All-Russia Research Institute of Agricultural Biotechnology, Timiryazevskaya 42, 127550 Moscow, Russia; alexandrov@iab.ac.ru (O.S.A.); divashuk@gmail.com (M.G.D.); razumovao@gmail.com (O.V.R.); uldas1508@gmail.com (D.S.U.); karlovg@gmail.com (G.I.K.)

**Keywords:** *Cannabis sativa* L., DNA repeat, 45S rDNA genes, intergenic spacer (IGS), physical mapping, fluorescent in situ hybridization (FISH), oligonucleotide probes

## Abstract

Hemp (*Cannabis sativa* L.) is a valuable crop and model plant for studying sex chromosomes. The scientific interest in the plant has led to its whole genome sequencing and the determination of its cytogenetic characteristics. A range of cytogenetic markers (subtelomeric repeat CS-1, 5S rDNA, and 45S rDNA) has been mapped onto hemp’s chromosomes by fluorescent in situ hybridization (FISH). In this study, another cytogenetic marker (the tandem repeat CS-237, with a 237 bp monomer) was found, studied, and localized on chromosomes by FISH. The signal distribution and karyotyping revealed that the CS-237 probe was localized in chromosome 6 with one hybridization site and in chromosome 8 with two hybridization sites, one of which colocalizes with the 45S rDNA probe (with which a nucleolus organizer region, NOR, was detected). A BLAST analysis of the genomic data and PCR experiments showed that the modified CS-237 monomers (delCS-237, 208 bp in size) were present in the intergenic spacers (IGSs) of hemp 45S rDNA monomers. Such a feature was firstly observed in Cannabaceae species. However, IGS-linked DNA repeats were found in several plant species of other families (Fabaceae, Solanaceae, and Asteraceae). This phenomenon is discussed in this article. The example of CS-237 may be useful for further studying the phenomenon as well as for the physical mapping of hemp chromosomes.

## 1. Introduction

Hemp (*Cannabis sativa* L.) has been one of the first plants to be cultivated by humans [1,2]. It is a valuable spinning and oilseed crop [3]. Hemp seeds are processed to produce high-quality oil used in the food and paint industries [4]. Hemp fiber is characterized by high strength and is resistant to prolonged exposure to water; therefore, it has long been widely used in the manufacture of rigging [5].

Botanically, *C*. *sativa* is a member of the Cannabaceae family together with *Humulus lupulus* L. and *Humulus japonicus* Siebold & Zucc. [6]. All these species are dioecious plants and have sex chromosomes. They have been used in a range of investigations dedicated to plant systems of sex chromosomes [7,8,9,10,11]. *C*. *sativa* has an XY system of sex chromosomes with a larger Y. The identification of the sex-determination mechanisms in *C. sativa* has a significant part in the production of this crop. For the production of medical hemp, female plants are used; unpollinated flowers contain more tetrahydrocannabinol, so the presence of male plants in the population is undesirable. When growing technical hemp, it is necessary to pollinate with pollen from the male flowers of monoecious plants, or male plants, to produce oil or seeds, but male plants do not produce seeds, so it is more economical to grow monoecious varieties [12]. In the production of some types of fiber, on the contrary, male plants can play an important role. At the same time, sex-determining genes can also affect fiber quality [13]. To date, there have been no complete and reliable assemblies of male cannabis genomes containing the Y chromosome, probably because female plants are more often required in production. Without a comparison of Y-chromosomes with each other at the genome level, it is difficult to understand the evolution of sex in this group [14], while understanding the mechanisms of the origin of sex would allow breeders to undertake a more directed selection process.

Hemp was karyotyped by DAPI/C-banding staining to provide chromosome measurements, and by fluorescence in situ hybridization (FISH) with probes for 45S rDNA (pTa71), 5S rDNA (pCT4.2), a subtelomeric repeat (CS-1), and the *Arabidopsis* telomere repeat [15]. FISH with the pTa71 (45S rDNA sequences from wheat [16]) probe revealed a signal on the NOR-bearing (nucleolus organizer region) arm of the single chromosome pair of male and female plants (chromosome 8) [15]. The pTa71 DNA probe from wheat has often been used to study 35S-rDNA-bearing regions in different plants. The FISH signals were detected in different plants because this probe includes conserved 18S, 5.8S, and 26S rRNA genes. These genes are organized as a tandemly repeated DNA [17]. The monomer of this repeat consists of 18S-ITS1 (internal transcribed spacer) –5.8S–ITS2–26S–IGS (intergenic spacer) sequences. The spacers ITS1, ITS2, and IGS are not conserved and often have species-specific features. Therefore, they are widely used in DNA barcoding [18,19].

One of the features of IGS is the presence of internal subrepeats, which have previously been described in different organisms [20,21,22,23,24,25]. In some of them, subrepeats from the IGS are also present in the genome as independent satellites; for example, such cases were found in some plants of the Fabaceae, Solanaceae, and Asteraceae families [23,24,25].

Satellite DNA (satDNA) forms a substantial part of eukaryotic genomes and is involved in the formation of heterochromatin in many plant and animal species [26,27]. SatDNA is composed of non-coding monomers that are repeated in tandem to form arrays of varying lengths and structures. Often, satellites are locally amplified and their localization can be detected in chromosomes using FISH [28,29,30]. SatDNA monomers are usually simple—that is, they consist of a single sequence that does not have a substructure inside—but complex satDNAs also exist. Their monomers consist of duplicated subunits, which can be either inverted or different due to the insertion of foreign DNA [26,31,32]. When the similarity between subunits is less than that between complex monomers, higher order repeats (HOR) are said to form [33,34,35]. SatDNA has traditionally been isolated by centrifugation in a CsCl gradient or the digestion of total DNA with endonuclease enzymes [36,37]. However, the development of next-generation sequencing (NGS) made it possible to obtain huge arrays of genetic data and to search satDNA using special bioinformatic tools, such as Tandem Repeat Finder and RepeatExplorer [38,39,40].

In this article, we report the discovery of the CS-237 tandem repeat in *C. sativa*, which is present in the genome both as an independent satellite and as a subrepeat inside the 45S rDNA IGS. Additionally, the CS-237 consists of complex monomers, which are formed by two subunits. The organization and possible evolutionary path of this DNA repeat are discussed.

## 2. Results

### 2.1. Search and Amplification of the CS-237 Repeat

**Figure 1:**
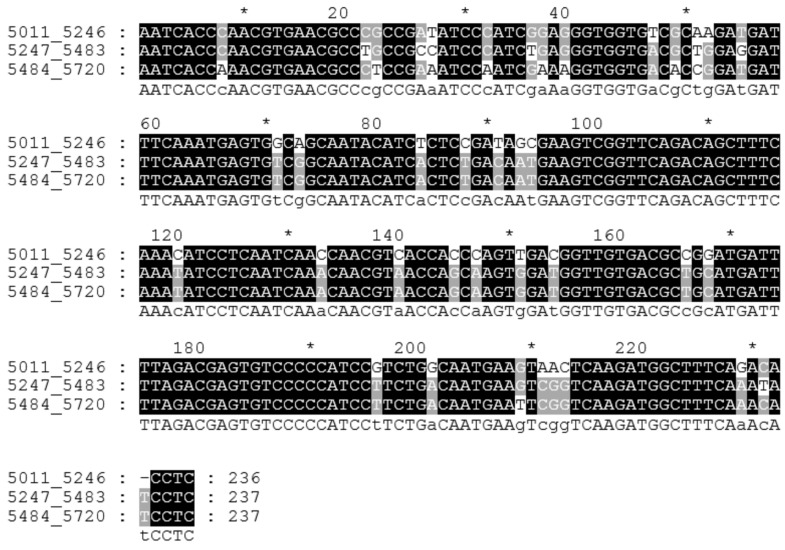
The alignment of the whole CS-237 monomers from scaffold 195. Codes of monomers consist of start_end nucleotide numbers in the scaffold.

In this study, the CS-237 tandem repeat was found in the Cannabis sativa genome using the software Tandem Repeat Finder (TRF). The first assembled region with this repeat was scaffold 195 from the canSat3 assembly [41]. TRF identified four tandemly repeated monomers in this scaffold (5011th–5246th bp, 5247th–5483rd bp, 5484th–5720th bp, and 5721st–5947th bp; the codes of the monomers consist of the start and end nucleotide numbers in the scaffold). The first three monomers are whole (236 bp, 237 bp, and 237 bp, respectively), and the last one is incomplete (227 bp). The whole monomers were aligned (Figure 1), and the alignment showed an identity level of about 85–94% (Table S1). Additionally, the CS-237 monomers were found in several other scaffolds from the canSat3 assembly (Table S2). The identity levels among all the collected monomers were similar to those in scaffold 195. 

**Figure 2:**
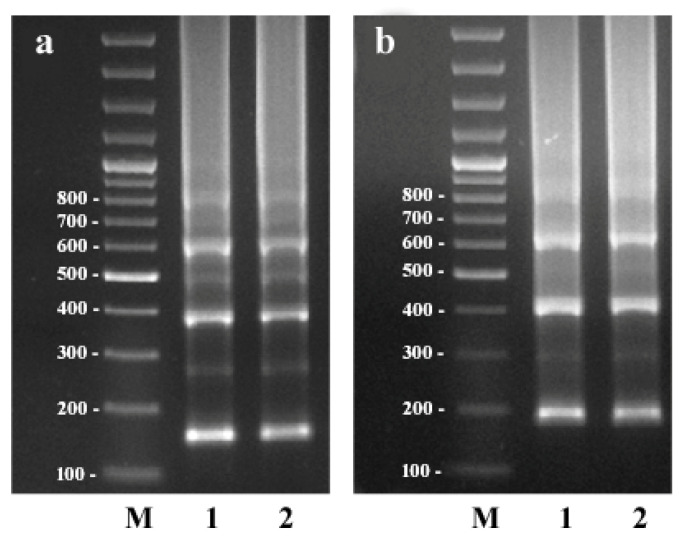
The results of PCR experiments with CS-237f/CS-237r1 (**a**) and CS-237f/CS-237r2 (**b**) primer pairs. **1**—C. sativa male; **2**—C. sativa female; **M**—molecular weight marker with 100 bp steps.

A consensus sequence of the CS-237 monomer from scaffold195 was used for primer design (Table S3, Figure S1). PCR with the CS-237f/CS-237r1 and CS-237f/CS-237r2 primer pairs produced the same results (Figure 2). Ladder-like patterns were observed in lanes corresponding to C. sativa DNA (the patterns of the male and female samples were similar). These patterns had about four major fragments, each of which differed from its neighbors by the length of the monomer. After the fourth monomer, the patterns turned into a smear. The patterns also contained minor fragments between the major ones. This may have indicated the presence of subunits inside the CS-237 monomers. To verify this, a BLAST self-comparison of the two CS-237 monomers was conducted, which showed that the CS-237 monomer included two subunits that were 111 and 126 bp in length. The alignment of these subunits showed about 69% identity between them (Figure S2 and Table S4).

PCR experiments with the CS-237f/CS-237r1 and CS-237f/CS-237r2 primers were also carried out using *Humulus lupulus* and *Humulus japonicus* DNA. Neither primer pair annealed on these DNA samples. FISH experiments with the CS-237 probe on the *H. lupulus* and *H. japonicus* chromosomes showed no signals. Additionally, the BLAST search of the CS-237 sequence (ON055366) in the *H. lupulus* genome showed that this repeat was absent. Thus, the CS-237 sequence is a species-specific DNA repeat for *C. sativa*.

### 2.2. Physical Mapping of the CS-237 Repeat with Other Cytogenetic Markers on C. sativa Chromosomes

**Figure 3:**
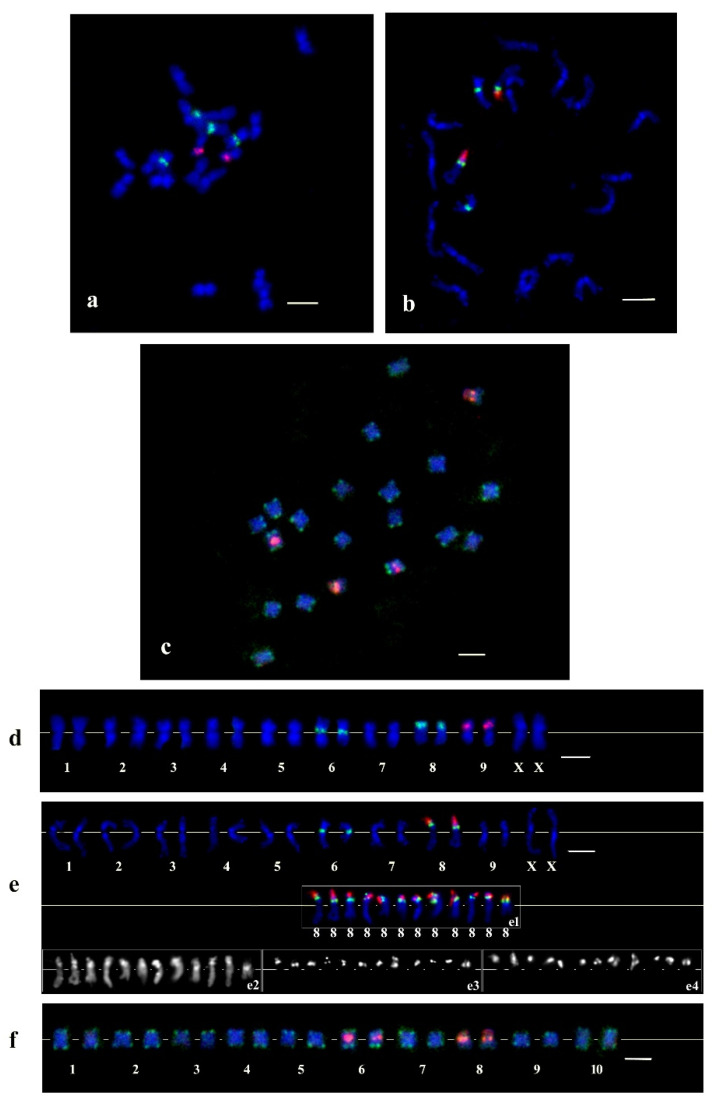
The results of FISH experiments and karyotyping with the CS-237 probe on C. sativa chromosomes: (**a**,**d**) green—CS237 probe, pink—5S rDNA probe; (**b**,**e**) green—CS237 probe, pink—45S rDNA; (**c**,**f**) green—CS-1 probe, pink—CS237 probe. Single channels: (**e2**) DAPI, (**e3**) CS237, (**e4**) 45S rDNA, and (**e1**) merged. Bar equals 5 μm.

The CS-237 tandem repeat probes were localized on the C. sativa chromosomes by FISH. In these experiments, four signals for the CS-237 were observed. The first pair of signals were located on the short arm of the metacentric chromosomes occupying the pericentromeric region (Figure 3). The second pair of signals was detected at the terminal part of the acrocentric chromosome’s short arm. In addition to these experiments, other cytogenetic probes (5S rDNA, 45S rDNA, and subtelomeric repeat CS-1) were localized on the same metaphase plates (Figure 3). These experiments showed that the CS-237 signals did not co-localize with 5S rDNA. The first pair of CS-237 signals was located on the chromosome pair with the signals of the subtelomeric repeat on both ends. Additionally, the second pair of CS-237 signals co-localized with the 45S rDNA signals. Karyotyping revealed that these CS-237 repeat signal pairs were located on chromosomes 6 and 8, respectively.

**Figure 4:**
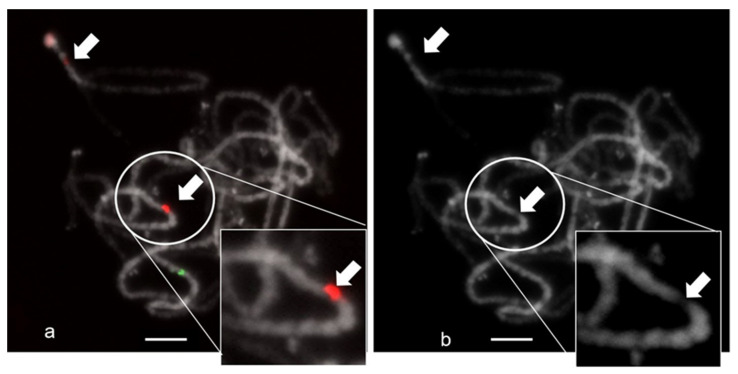
The results of FISH experiments with the CS-237 probe on pachytene C. sativa chromosomes: (**a**)—5S rDNA probe (green) and CS-237 probe (red); (**b**)—DAPI. Bar 5 μm. Arrows mark the localization sites with the euchromatin region.

FISH combined with 5S rDNA was performed at the pachytene stage of meiosis to more precisely estimate the CS-237 chromosome localization (Figure 4). Chromosomes in meiosis, and especially at the pachytene stage, are well differentiated and are longer than the mitotic chromosome, which allows for higher-resolution hybridization. The hybridization of pachytene chromosomes showed the localization of the discovered repeat on chromosomes 6 and 8 in the euchromatin region, which may evidence a structural function for the CS-237 in the genome. Furthermore, on chromosome 8, the two clearly defined localization sites were detected; one of them colocalized with the nucleolus organizer region (NOR).

### 2.3. Molecular Search of the CS-237 in 45S rDNA

**Figure 5:**
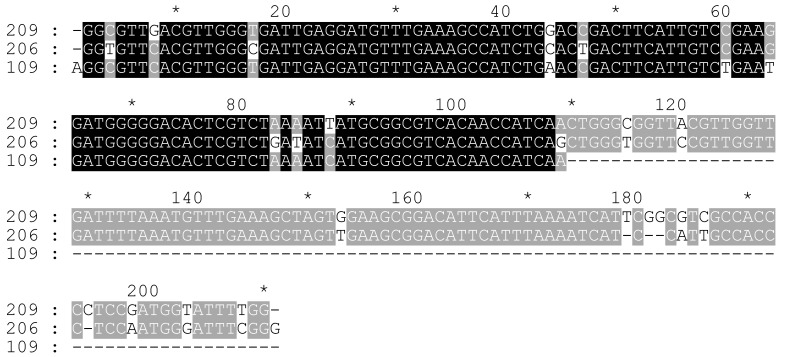
The alignment of the delCS-237 monomers from the 52978632nd–52979130th bp region of the WRXK01000002 contig.

To find the points of CS-237 and 45S rDNA co-localization, a common search of the CS-237 consensus sequence and ITS1–5.8S–ITS2 region (Y12587) was carried out by BLAST in the C. sativa “Whole-genome shotgun contigs” database. The top contig (WRXK01000002) contains the 52,978,632nd–52,979,130th bp region, which is very similar to CS-237. The analysis of the borders around this region showed that the CS-237-like repeat located in the intergenic spacer (IGS) between the 3′-end of the 26S gene and 5′-end of the 18S gene rDNA. The identified variant of the IGS sequence was 3595 bp in length and includes the CS-237-like region from the 2514th to 3037th nucleotides. The study of this IGS sequence using the TRF software revealed that the CS-237-like region consisted of about 2.5 monomers that were 209 bp (2514th–2722nd bp), 206 bp (2723rd–2928th bp), and 109 bp (2929th–3037th bp) in length, respectively. The TRF software presented a consensus sequence of the monomer for this repeat that was 208 bp in length (we named this sequence delCS-237). The collected delCS-237 monomers were aligned (Figure 5). The alignment revealed an identity between the first and second monomers of 89%. The 109 bp part of monomer showed 94% and 90% identity with the corresponding parts of the first and the second monomers, respectively (Table S5).

**Figure 6:**
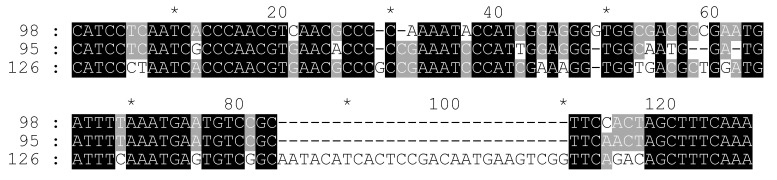
The alignment of short subunits of the delCS-237 monomers and 126 bp subunit of the CS-237 monomer.

**Figure 7:**
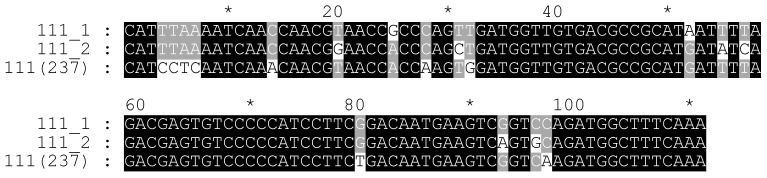
The alignment of long subunits of the delCS-237 monomers and 111 bp subunit of the CS-237 monomer.

A comparison of these monomers with the CS-237 monomer sequence showed that the delCS-237 monomers had a reverse orientation. Furthermore, the presence of subrepeats in the monomers was also detected. Thus, the first monomer had 98 and 111 bp subunits with a 55% level of identity (the alignment is presented in Figure S3). The second monomer had 95 and 111 bp subunits (the alignment is presented in Figure S4). Their level of identity was 50%. The short subunits of the monomers were very similar (85% level of identity). A comparison of those short subunits with the 126 bp part of the CS-237 revealed 62% and 61% identity. The alignment showed that the delCS-237 short subunits had a 27 bp deletion (AATACATCACTCCGACAATGAAGTCGG, Figure 6) or the 126 bp part of the CS-237 had the corresponding insertion. BLAST analysis revealed that this 27 bp region was highly similar to the 72nd–94th bp region of the 111 bp subunits. In turn, the 111 bp subunits showed high identity (92%) between themselves as well, with the 111 bp part of the CS-237 (90% and 86 %, respectively; the alignment is presented in Figure 7).

**Figure 8:**
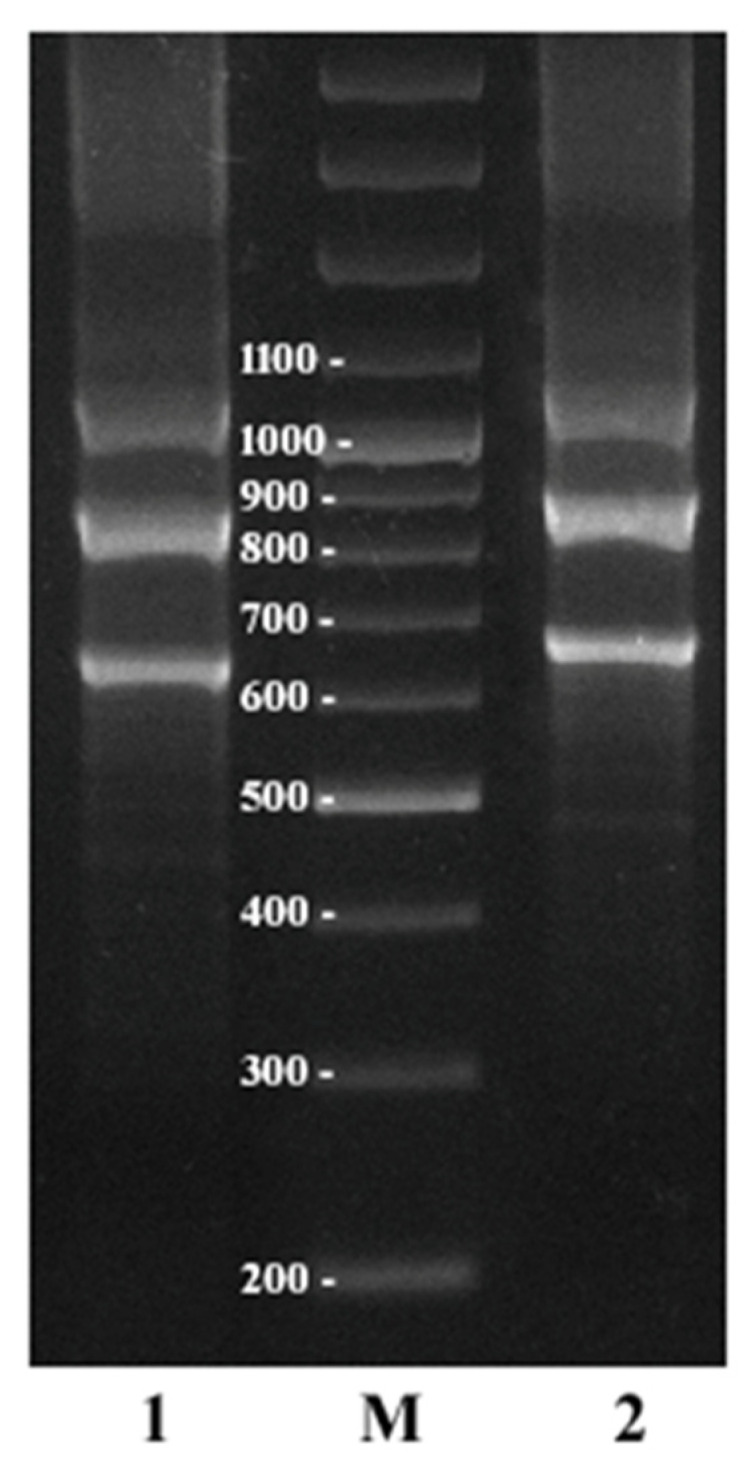
The results of PCR with CS-237r1/igs2004r (**1**) and CS-237r2/igs2004r (**2**) primer pairs in *C. sativa*. M—molecular weight marker with 100 bp steps.

The location and organization of the delCS-237 region inside the IGS were also studied by PCR experiments. PCR products were obtained with the CS-237r1/igs2004r and CS-237r2/igs2004r primer pairs (igs2004r is a reverse primer for the IGS’ amplification from [42]; it anneals on the 5′-ends of 18S rRNA genes). The PCR product consisted of three expected fragments that were about 620, 825, and 1035 bp in length for the first primer pair and about 660, 875, and 1080 bp for the second one (Figure 8). These results fully confirmed the conclusions about the co-localization of the delCS-237 and 45S rDNA described above. 

### 2.4. Additional Study of C. sativa IGS Variants

In 2004, Hsieh et al. [42] reported sequencing of the *C. sativa* IGS. Their sequence was 984 bp in length, which was very different from our results (we only found a 3595 bp IGS: the 52,978,632nd–52,979,130th bp region of the WRXK01000002 contig). BLAST analysis did not show the presence of the whole of Hsieh’s sequence in all the published assemblies of the *C. sativa* genome. The alignments did show that Hsieh’s sequence was very similar to the 1st–931st and 3565th–3595th regions of the 3595 bp IGS and does not include the delCS-237 monomers (Figure S5). Thus, Hsieh’s sequence has a 2611 bp deletion located between the 931st and 932nd nucleotides.

Additionally, we carried out BLAST alignment of the IGS sequence without the delCS-237 array against all the published assemblies of the *C. sativa* genome. The analysis showed that all the instances of the whole IGSs identified contained the delCS-237.

### 2.5. Bioinformatical Analysis of CS-237 and delCS-237 Repeats Localization in Chromosomes

The 90%+ identity alignment of the CS-237 and the delCS-237 repeats against the chromosome level assembly of *C. sativa* (GCA_016165845.1) (Table S6) shows that the CS-237 was present in tandem arrays of order-of-magnitude different lengths in chromosomes 6, 8, and X in regions 30–35 Mbp, 0.35–0.75 Mbp, and 54.65–54.67 Mbp, respectively. The delCS-237, however, was only present in the region 1.2–1.95 Mbp in chromosome 8, and aligned to the SRR10189115_Cannabis_sativa.1494100 read, which was only partially due to the highly similar subrepeats with the CS-237. The produced bit-scores never exceeded those of the CS-237 alignment at the same location.

The profiles (Figure S6) of CS-237′s high-scoring pairs (HSPs) implied the presence of large tandem arrays of (CS-237)n in chromosomes 6, 8, and X. The chromosome 6 and 8 (CS-237)n regions were especially similar including in the proportion of non-CS-237 insertions and their size distributions, and the X chromosome (CS-237)n region was too short to be detected with FISH.

delCS-237 repeats were found only in chromosome 8, and in a different context: the delCS-237 repeats were always in pairs (delCS-237)2 in the spacer between the 45S rDNA near the 18S sub-unit (Table S5). There were two configurations of spacers between rDNAs depending on their orientation: the common N-configuration, which had no similar proteins from the NCBI non-redundant protein sequences (nr) database and was found between co-oriented 45S rDNAs, and the rare P-configuration. The latter was found only twice in the long reads analyzed, but it is of particular interest, as it was shown to contain two reversed copies of a known uncharacterized protein (LOC110277282) flanking two copies of (delCS-237)2 (Figure S6).

Based on the total Cannbio-2 genome size of 914 Mbp and the BLAST results presented in Table S6 for both identity levels, the CS-237 and delCS-237 genome proportions can be estimated to lie within the ranges of 0.1–0.62% and 0.002–0.023%, respectively, depending on the strictness of the parameters.

## 3. Discussion

Repetitive DNA sequences, including tandem satellite DNA, may play an important role in the mechanisms of sex-chromosome differentiation in both animals and plants [43,44,45,46]. After recombination inhibition, the chromosome that does not exchange a site with a homolog (in the case of Cannabaceae, the Y chromosome) begins to accumulate repetitive sequences [46]. Large, bright, tandemly organized satellite DNA repeats can serve as good cytogenetic markers for the detection of sex chromosomes [47]. In the Cannabaceae *sensu stricto* family, the subtelomeric repeat helps to cytologically differentiate the sex chromosomes of hemp, common hop, and Japanese hop [9,10,15]. It is likely that these sequences, which have some homology, were directly involved in the reorganization of chromosomes during the phylogeny of this group. Molecular cytogenetic markers make it possible to identify a pseudoautosomal region and determine the orientation of the sex chromosomes in meiosis, which may be significant in the transfer of economically valuable traits in the selection process. Based on the morphology, hemp seed’s sex chromosomes can be classified as evolutionarily young chromosomes [7]; however, studies at the transcriptome level have shown that the cannabis Y chromosome is highly degenerate and is one of the most ancient in evolutionary terms [48]. The discovery of new species-specific repeat sequences, especially in such a conserved region as the ribosomal DNA region, sheds light on the phylogeny of this interesting group and the emergence of sex in it. In this regard, it may be of interest in the future to develop new markers, as well as to search for existing markers based on satellite DNA in the genomes of related Canabaceae *sensu lato* species, primarily in the genomes of monoecious and hermaphroditic *Parasponia* and *Trema*, as well as in the dioecious *Lozanella* [14].

In this article, we described our findings regarding the CS-237, IGS-like DNA tandem repeat in hemp. PCR experiments showed an absence of the hemp CS-237 monomers in the genomes of closely related species (*H. lupulus* and *H. japonicus*). Additionally, FISH experiments did not show any signals for *H. lupulus* and *H. japonocus* chromosomes with the CS-237 probes. These results indirectly testify that the CS-237 monomers do not form large tandem arrays in *H. lupulus* and *H. japonocus* as in *C. sativa*. Thus, the case with the CS-237 repeat does not follow the satellite DNA library theory [49,50]. According to this theory, monomers of the same satellite repeat are included in the genomes of closely related species but the numbers of these monomer copies may significantly differ. Such cases were previously described for a wide range of organisms, such as *Cucurbita* ssp. [51], *Triticeae* ssp. [52], *Chironomus* ssp. [53], and *Trichogramma* spp. [54]. However, the CS-237 is a species-specific repeat that does not have even single monomers in the genomes of closely related species (*Humulus* spp.).

FISH experiments on *C. sativa* chromosomes revealed that the CS-237 probe had two points of localization: as an independent satellite on chromosomes 6 and 8, and as a part of the 45S IGS on chromosome 8 (this variant had been named delCS-237, and it had a 208 bp consensus monomer). The results of the bioinformatical analysis fully support the results of the FISH experiments; chromosomes 6 and 8, in assembly, corresponded to the same chromosomes in our experiment. The monomers of both CS-237 and delCS-237 consisted of two subrepeats (that is, they were essentially high-order repeats, HORs) and showed polynucleotide differences in the one of subrepeats, and a high level of identity in the other. Overall, a similar situation was observed by Ruiz-Ruano et al. (2018) in *Pyrgomorpha conica* [32]. They reported satDNA superfamilies consisting of HOR families, which had monomers with non-homologous internal subrepeats. For example, the SF05 superfamily united six families with monomers that have a common 73 bp subrepeat. The monomers of four families also included other different subrepeats. Similarly, *C. sativa* essentially has two HORs (the CS-237 outside the 45S rDNA IGS and delCS-237in the 45S rDNA IGS) with a common 111 bp subrepeat. Thus, our results of the hemp CS-237 and delCS-237 investigation and the previously described results in grasshoppers show that a HOR formation with non-homologous internal subrepeats is a common scenario in satDNA evolution for plants and animals.

The presence of the CS-237 as an independent satellite and as a part of the IGS in the *C. sativa* genome is interesting and has not been widely observed. Previously, similar cases were described for some plants of the Fabaceae, Solanaceae, and Asteraceae families [23,24,25]. The authors of these investigations raised the question of the origins of these independent satellites and their IGS-linked copies, and two hypotheses were put forward. According to the first hypothesis, the independent satellites from other chromosomes invaded the rDNA unit, becoming an IGS subrepeat. Such conclusions were made in studies of *Phaseolus* ssp., *Vicia faba*, and *Chironomus* ssp. [55,56,57]. The second hypothesis suggested reverse events. That is, the independent satellites evolved from the corresponding IGS subrepeats. This opinion was shared by Macas et al. (2003), Lim et al. (2004), Jo et al. (2009), and others [23,24,58,59]. The range of the studied species in these cases also varied (*Vicia sativa*, *Solanum* spp., *Nicotiana* spp., and *Phaseolus* spp.). In *C. sativa*, an unclear situation with a preference for one of the proposed hypotheses was observed. If we assume that the events occurred according to the first hypothesis, then a 27 bp deletion should have occurred in the 126 bp subrepeat of CS-237 when the monomers were inserted into the IGS. On the other hand, if the second hypothesis is correct, then a migration of the monomers from the IGS to other chromosomes was accompanied by an insertion of the 27 bp region from the 111 bp subunit to the corresponding point of the 98 bp subunit. Both these events are equiprobable, since their molecular mechanisms have not been studied in detail. In addition, it cannot be ruled out that the pathways for the appearance of the IGS-linked satellites may be different, and both hypotheses are plausible.

Nevertheless, the question of how CS-237 and delCS-237 originated in *C. sativa* is not the only one; another is when the events associated with their appearance occurred. Since large arrays of the CS-237 are absent in *Humulus*, it can be assumed that its amplification in *C. sativa* occurred after the divergence of *Humulus* and *Cannabis*, that is, not earlier than 21 million years ago [15,60].

## 4. Conclusions

In *C. sativa* genome, the CS-237 species-specific tandem repeat was found using TRF. We have observed the FISH signals on chromosomes 6 and 8 with the CS-237 probe and have been able to correlate the reference chromosome assembly of *C. sativa* with physical chromosomes. Thus, we have obtained a new useful cytogenetic marker.

Additionally, we have discovered the co-localization of the CS-237 and 45S rDNA signals. The modified sequence of the CS-237 (delCS-237) was found inside the IGS of every 45S rDNA monomer. We found no exception across the carefully reviewed pool of hemp IGSs. However, Hsieh et al. (2004) previously reported that they had amplified and sequenced the shorter *C. sativa* IGS without delCS-237. The discrepancies of the lengths and sequences of the *C. sativa* IGSs between the studies could be related to the different plant materials and PCR approaches.

Finally, we hope that our findings will be helpful for the further study of the *C. sativa* genome and the nature of IGS-linked DNA repeats in general.

## 5. Materials and Methods

### 5.1. Plant Material and Bioinformatic Analysis

For the study of mitosis metaphase chromosomes, which have a well-defined structure, cv ‘‘Zenitsa’’ male and female seedlings of *C. sativa* were harvested (P.P. Lukyanenko Krasnodar Research and the Development Institute of Agriculture, Krasnodar, Russia). To study meiosis at the pachytene stage, which allowed for a higher resolution of hybridization, young male buds from the “T-80” line of *C. sativa* were provided by Dr. S. Dolgov (Branch of M. M. Shemyakin and Yu. A. Ovchinnikov Institute of Bioorganic Chemistry of the RAS, Pushchino, Moscow Region, Russia). The DNA repeat search in the *C. sativa* genome was carried out using the TRF software [39]. The GeneDoc software was used for manipulating the sequences, constructing alignments, and calculating the identity levels [61].

The chromosome-level assembly of *Cannabis sativa* L. (GenBank: GCA_016165845.1) [62] was used to detect loci of both CS-237 and delCS-237 by aligning them against chromosomes with blastn from the NCBI BLAST+ package [63] with the parameters -task blastn -no_greedy -evalue 0.01 -word_size 10 -qcov_hsp_perc 95 -perc_identity 85. 

Long reads from a PacBio scan of *C. sativa* L. (Accession: PRJNA562042, SRA: SRR10189115) [64] were checked for both CS-237 and delCS-237 using gmapl (http://research-pub.gene.com/gmap/ accessed on 30 March 2022) [65] with the parameters –min-identity = 0.7 --nosplicing –no-chimeras. The reads that contained either repeat were extracted and aligned with reference 45S rDNA (GenBank: KM036288.1, *Oryza nivara* 45S rDNA).

### 5.2. Chromosome Preparation

Actively growing root tips approximately 1.5–2.0 cm long were harvested separately from young hemp seedlings and immediately pretreated with an aqueous solution of 2 mM 8-hydroxyquinoline for 2 h at room temperature (RT), and then for 2 h at 4 °C in the dark. An ethanol/glacial acetic acid (3:1 v/v) mixture was used for fixation. Meristems 2 mm long were cut from the fixed root tips and digested in a 10 mL enzyme solution (0.5% cellulase Onozuka R-10 (Serva, Germany) and 0.5% pectolyase Y-23 (Seishin Corp., Kobe, Japan) in a 10 mM citrate buffer (pH = 4.9)) for 1.5 h at +37 °C. Suspended cells were used for mitotic chromosome preparation as described by Kirov et al. [66]. Meiotic chromosome preparations were made from young flower buds as described by Divashuk et al., 2014 [15].

### 5.3. DNA Isolation

DNA isolation was performed as described by Doyle and Doyle [67] with some modifications. The extraction buffer contained 100 mM Tris-HCl (pH = 8.0), 20 mM EDTA (pH = 8.0), 2 M NaCl, 1.5% CTAB, 1.5% PVP, and 0.2% β-mercaptoethanol. A 15 mM ammonium acetate solution in 75% ethanol was used for DNA washing.

### 5.4. PCR Analysis

The repeats were PCR-amplified using specific primers (Table S3) that were developed using the Primer3 software [68] or found in the paper [42]. The PCR program included the following steps: 94 °C for 5 min; 35 cycles of 94 °C for 20 s, N °C for 20 s (where N is the annealing temperature for a given primer pair from Table S3) and 72 °C for 1 min, and 72 °C for 10 min. The PCR results were obtained by electrophoresis through a 1.5% agarose gel at 6 V/cm for 1 h.

### 5.5. DNA Probes and Fluorescent In Situ Hybridization (FISH)

The following probes were used: pTa71 (18S-28S rDNA) [11], pCT4.2 (5S rDNA) [69], the CS-1 probe (*C. sativa* subtelomeric repeat JX402748), and the CS-237 probe (*C. sativa* repeat ON055366). CS-1 (a clone preserved from the research by Divashuk et al., 2014), 5S rDNA, and 18S-28S rDNA were labeled by nick-translation with digoxigenin-11-dUTP, and CS-237 was labeled by PCR with biotin-16-dUTP according to the manufacturer’s instructions (Boehringer, Ingelheim am Rhein, Germany). The FISH experiments were performed as described by Karlov et al. [70]. The stringency of the FISH was about 72% (washing conditions: 15 min in 0.1× SSC at 42 °C). The chromosomes were counterstained with 1 mg/mL DAPI and mounted in Vectashild (Vector Laboratories, UK). An AxioImager M1 fluorescent microscope (Zeiss, Oberkochen, Germany) was used to observe the chromosome preparations. The metaphase plates with fluorescent signals were photographed with a monochrome AxioCam MRm CCD camera and visualized using the Axiovision software (Zeiss). The metaphase chromosomes were classified according to Levan et al. [71] based on their arm ratios and FISH hybridization patterns.

## Data Availability

Not applicable.

## Supplementary Materials

The following supporting information can be downloaded at https://www.mdpi.com/article/10.3390/plants11111396/s1. Figure S1: The CS-237-based primers and their locations, Figure S2: The alignment of the 126 bp and 111 bp subunits of the CS-237 consensus monomer, Figure S3: The alignment of the 98 bp and 111 bp subunits of the first delCS-237 monomer from the 52,978,632nd–52,979,130th bp region of the WRXK01000002 contig, Figure S4: The alignment of the 95 bp and 111 bp subunits of the second delCS-237 monomer from the 52,978,632nd–52,979,130th bp region of the WRXK01000002 contig, Figure S5: The alignment of the 3595 bp IGS (52,978,632nd–52,979,130th bp region of the WRXK01000002 contig, IGS long) and the 984 bp IGS from Hsieh et al. 2004 (IGS short) [42], Figure S6: Characteristics of (CS-237)n and (delCS-237)2 repeat clusters in the different genomic regions. The colors in the BLASTN alignments represent the bit-scores, with red highlighting bit-scores >= 200; pink, 80–200; green, 50–80, and blue, 40–50, Table S1: Levels of identity between the CS-237 monomers from scaffold 195, which were calculated using the GeneDoc software based on the alignment (see Figure 1), Table S2: The results of the BLAST search of the CS-237 sequences in the canSat3 assembly of the *C. sativa* L. genome, Table S3: The used primers and their parameters, Table S4: Levels of identity between the 126 bp and 111 bp subunits of the CS-237 consensus monomer, which were calculated using the GeneDoc software based on the alignment (see Figure S2), Table S5: Levels of identity between the delCS-237 monomers from the 52,978,632nd–52,979,130th bp region of the WRXK01000002 contig, which were calculated using the GeneDoc software based on the alignment (see Figure 4), Table S6: Quantity and localization of HSPs for CS-237 and delCS-237 repeats based on BLAST alignment against the chromosome-level assembly of *C. sativa* (GCA_016165845.1).
