# Supplementary material for: Study and Physical Mapping of the Species-Specific Tandem Repeat CS-237 Linked with 45S Ribosomal DNA Intergenic Spacer in Cannabis sativa L
Source: Plants (Basel). 2022 May 24;11(11):1396. doi: 10.3390/plants11111396 (PMC9183113; doi:10.3390/plants11111396)
Supplement: Supplementary file 1 [file plants-11-01396-s001.zip › Figures ed.pdf]

```
                   *        20         *        40   CS237-f ►        60
CS-237 : AATCACCCAACGTGAACGCCCGCCGAAATCCCATCGAAAGGTGGTGACGCTGGATGATTTC

                   *        80         *       100         *       120
CS-237 : AAATGAGTGTCGGCAATACATCACTCCGACAATGAAGTCGGTTCAGACAGCTTTCAAACAT

                   *       140         *       160         *       180
CS-237 : CCTCAATCAAACAACGTAACCACCAAGTGGATGGTTGTGACGCCGCATGATTTTAGACGAG

         ◄ CS237-1r     200        *  ◁ CS237-2r         *
CS-237 : TGTCCCCCATCCTTCTGACAATGAAGTCGGTCAAGATGGCTTTCAAACATCCTC : 237
```

Figure S1. The CS-237-based primers and their locations.

```
                  *         20         *         40         *         60
126 : CATCCCTAATCACCCAACGTGAACGCCCGCCGAAATCCCATCGAAAGGTGGTGACGCTGGATGA
111 : CATCCTCAATCAAACAACGTAA----CCACC-AAGT-------GGATGGTTGTGACGCCGCATGA

                  *         80         *        100         *        120
126 : TTTCAAATGAGTGTCGGCAATACATCACTCCGACAATGAAGTCGGTTCAGACAGCTTTCAAA
111 : TTTTAGACGAGTGTCCCC----CATCCTTCTGACAATGAAGTCGGTCAAGATGGCTTTCAAA
```

Figure S2. The alignment of the 126 bp and 111 bp subunits of the CS-237 consensus monomer.

```
                  *         20         *         40         *         60
 98 : CATCCTCAATCACCCAACGTCAACGCCCCAAAATACCATCGGAGGGGTGGCGACGCCGAATGAT
111 : CATTTAAAATCAACCAACGTAACCGCCC--AGTTG--AT------GGTTGTGACGCCGCATAAT

                  *         80         *        100         *        120
 98 : TTTAAATGAATGTCCGCT----------------------TCCA-CTAGCTTTCAAA
111 : TTTAGACGAGTGTCCCCCATCCTTCGGACAATGAAGTCGGTCCAGATGGCTTTCAAA
```

Figure S3. The alignment of the 98 bp and 111 bp subunits of the first delCS-237 monomer from the 52,978,632nd–52,979,130th bp region of the WRXK01000002 contig.

```
                  *         20         *         40         *         60
 95 : CATCCTCAATCGCCCAACGTGAACACCCCGAAATCCCATTGGAGGGTGGCAATGGATGATTTT
111 : CATTTAAAATCAACCAACG-GAACCACCCAG-----CTGATGGTTG-TGACGCCGCATGATATC

                  *         80         *        100         *
 95 : AAATGAATGTCCGC-----------------------TTCAACTAGCTTTCAAA
111 : AGACGAGTGTCCCCCATCCTTCGGACAATGAAGTCAGTGCAGATGGCTTTCAAA
```

Figure S4. The alignment of the 95 bp and 111 bp subunits of the second delCS-237 monomer from the 52,978,632nd–52,979,130th bp region of the WRXK01000002 contig.

```
                          *         20         *         40         *
 IGS long  : TCCCTCCCCCTTTTTTATAACAACACACTTTCAAGGCCTGAGGTTATCAAGGTGTTTG
 IGS short : TCCCTCCCCCTTTTTCATAACAACACACTT-CAAGGCCTGAGGT-ATCAAGGTGTT-G

             60         *         80         *        100         *
 IGS long  : TGGTGCTTCCATCAACACAAGTTGCGGCCGTAGCAATAACATATGAGCTCTTTCCCTT
 IGS short : TGGTGCTTCCATCAACACAAGTTGCGGCCGTAGCAATAACATATGAGCTCTTTCCCTT

              120         *        140         *        160         *
 IGS long  : TGGACTTGATATGCAAGCCAAAGTGTTGGTCGGGTTCAACAAAAGACTTAGTCCGAA
 IGS short : TGGACTTGATATGCAAGCCAAAGTGTTGGTCGGGTTCAACAAAAGACTTAGTCCGAA
```

```
            180         *        200         *        220         *
 IGS long : AAATAATTTTTTCCTGTGTTGTTCAATAATATGTGTCCTCTTTCCCTTTGGGACTTGA
IGS short : AAATAATTTTTTCCTGTGTTGTTCAATAATATGTGTCCTCTTTCCCTTTGGGACTTGA

              240         *        260         *        280         *
 IGS long : TATGAGCCTCAAAAGTGTTGGTCGGGAACAACAAAAGACTTAGACCGAAAAATAATTT
IGS short : TATGAGCCTCAAAAGTGTTGGTCGGGAACAACAAAAGACTTAGACCGAAAAATAATTT

                300         *        320         *        340
 IGS long : TTTTCTGTGCTGTTGATATAGGTGTCCATGTACCCAGTTTGGCATTGGTGCACCAAAG
IGS short : TTTTCTGTGCTGTTGATATAGGTGTCCATGTACCCAGTTTGGCATTGGTGCACCAAAG

             *        360         *        380         *        400
 IGS long : TATGTGGGCTTTCATGCCTAAGTTCACCACACTCCCATTCACTGCCTTGGCGCACCAC
IGS short : TATGTGGGCTTTCATGCCTAAGTTCACCACACTCCCATTCACTGCCTTGGCGCACCAC

               *        420         *        440         *        460
 IGS long : GCTTGAATGAACACGCATCACAAAAAGGTTATCACGCCCCATTACGGAACCCTTGTTG
IGS short : GCTTGAATGAACACGCATCACAAAAAGGTTATCACGCCC-ATTACGGAACCCTTGTTG

                 *        480         *        500         *        520
 IGS long : TGCGGTTATCACACGGCCTTGTTGTCACTTGGTGCGCTAAGAGTGGGTGCACAGTGCC
IGS short : TGCGGTTATGACACGGCCTTGTTGTCACTTGGTGCGCTAAGAGTGGGTGCACAGTGCC

                   *        540         *        560         *        580
 IGS long : CTTGTTGTGCCTTGGTGCAGTAAGAGTGGGTGCACATATGTCTACGGAGAAAAAATCA
IGS short : CTTGTTGTGCCTTGGTGCAGTAAGAGTGGGTGCACATATGTCTACAGAGAAAAATTCA

                     *        600         *        620         *        6
 IGS long : TCTATGTTCCTGGCAGAGGCTTAAAAGCCCATATTGCTCTTTAGGGGGGTGAGCTGGG
IGS short : TCTATGTTCCTGGCAGAGGCTTAAAAGCCCATATTGCTCTTTAGGGGGGTGAGCTGGG

            40         *        660         *        680         *
 IGS long : AGGGTCACCACACTGCACCCACACGTGAGGTTAACTGACGCCCTTGTGGTGCCTTGGT
IGS short : AGGGTCACCACACTGCACCCACACGTGAGGTTAACTGACGCCCTTGTTTGTGCCTTGGT

             700         *        720         *        740         *
 IGS long : GCGCTAAGAGTGGGTGCACATTGTCCTTGTTGTGCCTTGGTGCGCTAAGAGGGGGTGC
IGS short : GCGCTAAGAGTGGGTGCACATTGCCCTTGTTGTGCCTTGGTGCGCTAAGAGTGGGTGC

              760         *        780         *        800         *
 IGS long : ACAATGCACCCGCAAGTGAGGTTAACAGAGACCCTTCTTGTGCCTTGGTGCACTAAGA
IGS short : ACAATGCACCCGCAAGTGAGGTTAACAGAGACCCTTCTTGTGCCTTGGTGCACTAAGA

              820         *        840         *        860         *
 IGS long : GTGGGTGCACATTGGACTAAGAGTGGGTGCACAGTGCACCCACAAGTGAGGTTAACAG
IGS short : GTGTGTGCACATTGGACTAAGAGTGGGTGCACAGTGCACCCACAAGTGAGGTTAACAG

                880         *        900         *        920
 IGS long : AGGCCCTTGTTGTGGCTTGGTGCACTGAGAGTGTGTGCACAGTGCACCCACATGTGAT
IGS short : AGGCCCTTGTTGTGCCTTGGTGCACTGAGAGTGTGTGCACAGTGCACCCACATGTGAT

             *        940         *        960         *        980
 IGS long : GATAAGAGTGGCCCTTGTTGTGCCTTGGTGCACTAAGAGTGGGTGCACAGTGCACCCA
IGS short : GAT-------------------------------------------------------

               *       1000         *       1020         *       1040
 IGS long : CAAGTGAGGTTAACAGAGGCCCTTGTTGTGCCTTGGTGCACTGAGAGTGTGTGCACAG
IGS short : ----------------------------------------------------------

                 *       1060         *       1080         *       1100
 IGS long : TGCACCCACATGTGATGATAAGAGTGGCCCTTGTTGTGCCTTGGTGCACTAAGAGTGG
IGS short : ----------------------------------------------------------
```

```
                   *      1120         *      1140         *      1160
 IGS long : GTGCACAGTGCACCCACAAGTGAGGTTAACAGAGGCCCTTGTTGTTCCTCGGTGCACT
IGS short : ----------------------------------------------------------

                     *      1180         *      1200         *      12
 IGS long : AAGAGTGTGTGCACAGTGCACCCTCAAGTGAGGATAAGAGAGGCCCTTGTTGTGCCTT
IGS short : ----------------------------------------------------------

            20         *      1240         *      1260         *
 IGS long : GGTGCACTAAGAGTGGGTGCACAGTGCACCCTCAAGTGAGGATAAGAGAGGCCCTTGT
IGS short : ----------------------------------------------------------

            1280         *      1300         *      1320         *
 IGS long : TGTGCCTTGGTGCACTAAGAGTGGGTGCACAGTGCACAAGCAACGCCCGGGCAAAACT
IGS short : ----------------------------------------------------------

              1340         *      1360         *      1380         *
 IGS long : TTGAATTTATCCATGAGATTTTGCACAGTGCTTAGACACATTAAAAAAATTGTGGATA
IGS short : ----------------------------------------------------------

                1400         *      1420         *      1440         *
 IGS long : TCAATTTTCATGCCAAAAATGTTAGTATATTTTTTTTTTATGATTTTTTCCCTTCTAA
IGS short : ----------------------------------------------------------

                  1460         *      1480         *      1500
 IGS long : CTAGTTAAAAAATTATTAAAAAAAAATATACTAATCATTTTGTCATGAAAATTGATATC
IGS short : ----------------------------------------------------------

             *      1520         *      1540         *      1560
 IGS long : AACACCTTTTATTATGTGTCTAACCACTGTGTAAAATCTCATGGAAGAATTCATAGTT
IGS short : ----------------------------------------------------------

               *      1580         *      1600         *      1620
 IGS long : CACACCCATGGTGCGCACTCGTGGAAATCCACCAAGTGCACAACAAACGCCCAAGCAA
IGS short : ----------------------------------------------------------

                 *      1640         *      1660         *      1680
 IGS long : AACTATGAATTTTTCCGTGAGATTTTGCACAGTGCTTAGACACATTAAAAAATGGTGG
IGS short : ----------------------------------------------------------


 IGS long : ATATCAATTTTCATGCCAAAAAGATTAGTATATTTTTTTTTTATGATTTTTTCCCTTC
IGS short : ----------------------------------------------------------

                   *      1700         *      1720         *      1740
 IGS long : TAACTAGTTAAAAAATTATTAAAAAAAAATATACTAATCATTTTGGCATGAAAATTGAT
IGS short : ----------------------------------------------------------

                     *      1760         *      1780         *      18
 IGS long : ATCAACACCTTTTATTATGTGTCTAACCACTGTGCAAAATCTCATGGAAGAATTCATA
IGS short : ----------------------------------------------------------

            00         *      1820         *      1840         *
 IGS long : GTTCACACCCATGGTGCGCACTCGTGGAAATCCACCAAGTGCACAACAAACGCCCGAG
IGS short : ----------------------------------------------------------

            1860         *      1880         *      1900         *
 IGS long : CAAAATTATGAATTTCTCCGTGAGATTTTGCACAGTGCTTAGACACATAATAAAAGGT
IGS short : ----------------------------------------------------------

              1920         *      1940         *      1960         *
 IGS long : GTGGATATCAATTTTCATGCCAAAATGATTAGTATATTTTTTTTTTATGATTTTTTTCC
IGS short : ----------------------------------------------------------
```

```
                1980         *      2000         *      2020         *
 IGS long : TTTTTTTGGCAAAAAAATTATATAAAAAAAAATATTAATCTTTTTTTCATGAAAATTA
IGS short : ----------------------------------------------------------

                  2040         *      2060         *      2080
 IGS long : GTCTCCACGCGTTTTTTTATATGTCCAACCACCCTGCAAAATTTCATGGAAAAACTCC
IGS short : ----------------------------------------------------------

             *      2100         *      2120         *      2140
 IGS long : AAGTATTTCTTATACTTCCTTCACATATGTCTACAGAGAAAAAATCATCTATGTTCCT
IGS short : ----------------------------------------------------------

               *      2160         *      2180         *      2200
 IGS long : GGCAGAGCCTTAAAACCTCATATTGCTCTTTAGGGGGGTGAGCTGGGATGGGCAGGCA
IGS short : ----------------------------------------------------------

                 *      2220         *      2240         *      2260
 IGS long : AGGTCACCATGGGGAGGGCGCTGGGCAGGCCCTCTAACCCCCCTCCATGGTGCGCAGG
IGS short : ----------------------------------------------------------

                   *      2280         *      2300         *      2320
 IGS long : CTAGGCAAATTGGGCGTGGCAAAGGGTGATTAAGGGTGTTTCAAAGCCATCGTGACCG
IGS short : ----------------------------------------------------------

                     *      2340         *      2360         *      23
 IGS long : ACTTCGAGTCCGGACGAGCGATGGTGGCGACTCATCTGAAATTTTCCAGCGTCACAAG
IGS short : ----------------------------------------------------------

            80         *      2400         *      2420         *
 IGS long : GCTCCGCTAGCCCGCAGTGGGTGCCCGCGCCGCATGATTTTAAACGAGTGATTTTAAT
IGS short : ----------------------------------------------------------

            2440         *      2460         *      2480         *
 IGS long : CTCTCGTTTAAAATCATGCGGCGTTGACGTTGGGTGATTGAGGATGTTTGAAAGCCAT
IGS short : ----------------------------------------------------------

              2500         *      2520         *      2540         *
 IGS long : CTGGACCGACTTCATTGTCCGAAGGATGGGGGACACTCGTCTAAAATTATGCGGCGTC
IGS short : ----------------------------------------------------------

                2560         *      2580         *      2600         *
 IGS long : ACAACCATCAACTGGGCGGTTACGTTGGTTGATTTTAAATGTTTGAAAGCTAGTGGAA
IGS short : ----------------------------------------------------------

                  2620         *      2640         *      2660
 IGS long : GCGGACATTCATTTAAAATCATTCGGCGTCGCCACCCCTCCGATGGTATTTTGGGGTG
IGS short : ----------------------------------------------------------

             *      2680         *      2700         *      2720
 IGS long : TTCACGTTGGGCGATTGAGGATGTTTGAAAGCCATCTGCACTGACTTCATTGTCCGAA
IGS short : ----------------------------------------------------------

               *      2740         *      2760         *      2780
 IGS long : GGATGGGGGACACTCGTCTGATATCATGCGGCGTCACAACCATCAGCTGGGTGGTTCC
IGS short : ----------------------------------------------------------

                 *      2800         *      2820         *      2840
 IGS long : GTTGGTTGATTTTAAATGTTTGAAAGCTAGTTGAAGCGGACATTCATTTAAAATCATC
IGS short : ----------------------------------------------------------

                   *      2860         *      2880         *      2900
 IGS long : CATTGCCACCCTCCAATGGGATTTCGGGAGGCGTTCACGTTGGGTGATTGAGGATGTT
IGS short : ----------------------------------------------------------
```

```
                     *      2920         *      2940         *      29
 IGS long : TGAAAGCCATCTGAACCGACTTCATTGTCTGAATGATGGGGGACACTCGTCTAAAATC
IGS short : ----------------------------------------------------------

            60         *      2980         *      3000         *
 IGS long : ATGCGGCGTCACAACCATCAATTTCGGTGGGCGGTTACTTTCACACCTAGCGGTGCGG
IGS short : ----------------------------------------------------------

            3020         *      3040         *      3060         *
 IGS long : TCATGGGGTTTTTAAGGGCGTTTGTAAGCTTTGTCTTTCGACTTTATTGTCTGGGCAA
IGS short : ----------------------------------------------------------

              3080         *      3100         *      3120         *
 IGS long : GGTACGCAATCGTTTGAAATTATCTTGTGGCTAGCGTACGTGATAGTGTATGAGTGGT
IGS short : ----------------------------------------------------------

                3140         *      3160         *      3180         *
 IGS long : TTTTTGGTTGATTGTGTTGGTAGGCTCCGTGCTCGTGCATCGAACTACCAATCCTCCA
IGS short : ----------------------------------------------------------

                  3200         *      3220         *      3240
 IGS long : ACCTTCTTCAGGGTTGCTACAAGAGCGCTGCTCATTTGAGCGCAATGCGGTTTCCTGT
IGS short : ----------------------------------------------------------

             *      3260         *      3280         *      3300
 IGS long : GTTTGCTACCTCGGACGGAATGATTCATTTGGTTGCCTTTTTCCTCCTTTGTGGCTGT
IGS short : ----------------------------------------------------------

               *      3320         *      3340         *      3360
 IGS long : AATGGCTGCAAGGGGGACCTCGTAGCAGTCCTCGTGTCCCGAACGTGCCCTTCGTTAT
IGS short : ----------------------------------------------------------

                 *      3380         *      3400         *      3420
 IGS long : TGATTGTGTCGTTTCGGTCCTCGAGTGCCTGCTTGATCTCTCGGATGCGGAACGCTAC
IGS short : ----------------------------------------------------------

                   *      3440         *      3460         *      3480
 IGS long : GCGAGTGTGGGGGTCTGTGACCTTCGAACACTCAAATTTTCCATTTAAACGGATGACG
IGS short : ----------------------------------------------------------

                     *      3500         *      3520         *
 IGS long : TTCGTGGGGTGTACTGATCGTATGATCTGCATCTTGCGGCGTCCCCGAATGAGTGCT
IGS short : --------------------------CTGCATCTTGCGGCGTCCCCGAATGAGTGCT
```

Figure S5. The alignment of the 3595 bp IGS (52,978,632nd–52,979,130th bp region of the WRXK01000002 contig, IGS long) and the 984 bp IGS from Hsieh et al. 2004 (IGS short) [42].

Chr6: 30-35Mbp
~62% **(CS-237)n**
12867 HSPs with CS-237
91% repeat pairs without spacers
7.7% with spacers 100-1000bp
1.2% with spacers 1-10Kbp
0.1% with spacers 10-400Kbp
respective PacBio read: SRR10189115_Cannabis_sativa.1494100

Chr8: 0.35-0.75Mbp
~55% **(CS-237)n**
1007 HSPs with CS-237
90% repeat pairs without spacers
7.8% with spacers 100-1000bp
1.1% with spacers 1-10Kbp
0.1% with spacers 10-500Kbp
respective PacBio read: SRR10189115_Cannabis_sativa.1494100

ChrX: 54,65-54,67Mbp
~76,5% **(CS-237)n**
77 HSPs with CS-237
87% repeat pairs without spacers
13% with spacers 100-1000bp
0% with spacers 1-10Kbp
0% with spacers 10-500Kbp
respective PacBio read: SRR10189115_Cannabis_sativa.1494100

Chr8: 1.21-1.95Mbp
~2.3% **(delCS-237)2**
149 HSPs with CS-237
37% repeat pairs without spacers
13% with spacers 100-1000bp
37% with spacers 1-10Kbp
13% with spacers 10-500Kbp
respective PacBio read: SRR10189115_Cannabis_sativa.3007106

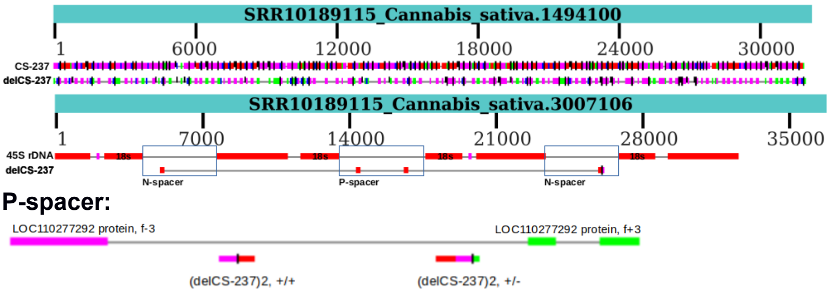

Figure S6. Characteristics of (CS-237)n and (delCS-237)2 repeat clusters in the different genomic regions. The colors in blastn alignments represent bit-scores, with red staying for bit-scores >=200, pink - 80-200, green – 50-80 and blue 40-50
