# Supplementary material for: Study and Physical Mapping of the Species-Specific Tandem Repeat CS-237 Linked with 45S Ribosomal DNA Intergenic Spacer in Cannabis sativa L
Source: Plants (Basel). 2022 May 24;11(11):1396. doi: 10.3390/plants11111396 (PMC9183113; doi:10.3390/plants11111396)
Supplement: Supplementary file 1 [file plants-11-01396-s001.zip › Tables ed.pdf]

Table S1. Levels of identity between the CS-237 monomers from scaffold 195, which were calculated using the GeneDoc software based on the alignment (see **Figure 1**).

Codes	5011_5246	5247_5483	5484_5720
**5011_5246**	100%	86%	85%
**5247_5483**		100%	94%
**5484_5720**			100%

Table S2. The results of the BLAST search of the CS-237 sequences in the canSat3 assembly of the *C. sativa* L. genome.

Sequence	Strand	Start	End	Span
scaffold195	+	5484	5720	237
scaffold17486	+	7388	8098	711
scaffold17486	+	6440	6913	474
scaffold13470	+	1008	2551	1544
scaffold4296	+	38023	43897	5875
scaffold20912	+	117	353	237
scaffold3525	-	3607	7190	3584
scaffold3525	-	2320	2556	237
scaffold14078	+	6777	7013	237
scaffold13470	+	2078	2314	237
scaffold22934	+	5157	5439	283
scaffold4296	+	66593	66829	237
scaffold17956	+	983	1311	329
scaffold28766	-	7551	8267	717
scaffold10734	-	6861	8876	2016
scaffold4296	+	32034	35391	3358
scaffold22858	+	1484	1900	417
scaffold73647	-	130	365	236
scaffold37689	-	1099	1333	235
scaffold4296	+	40623	40858	236
scaffold4296	+	40387	40622	236
scaffold4296	+	39915	40150	236
scaffold4296	+	39679	39914	236
scaffold83421	-	88	1194	1107
scaffold21990	-	27439	27911	473
scaffold14405	-	3129	3489	361
scaffold30934	+	461	1134	674
scaffold33591	-	905	1141	237
scaffold10734	-	3557	3793	237
scaffold22933	+	1	237	237
scaffold16081	+	278	514	237
scaffold13470	+	8196	8432	237
scaffold5339	-	2992	3228	237
scaffold10440	-	2268	2741	474
scaffold4296	+	5266	27122	21857
scaffold4296	+	4904	5501	598
scaffold20180	+	100	440	341
scaffold17486	+	6914	7624	711

scaffold20767	-	4716	4952	237
scaffold69780	+	562	797	236
scaffold4296	+	70899	71135	237
scaffold23728	+	4682	4918	237
scaffold19485	+	22	729	708
scaffold14773	+	977	7689	6713
scaffold13470	+	1248	1728	481
scaffold12450	+	285	521	237
scaffold85237	-	1289	2124	836
scaffold39593	-	2231	2467	237
scaffold10734	-	2958	3320	363
scaffold4400	+	18100	18460	361
scaffold4296	+	39205	39674	470
scaffold4296	+	3066	3602	537
scaffold3525	+	12505	12976	472
scaffold8890	-	14190	14426	237
scaffold547	-	7682	8029	348
scaffold33166	-	1944	2180	237
scaffold28541	-	539	775	237
scaffold10662	-	2526	9217	6692
scaffold4544	+	2706	2942	237
scaffold25614	+	1647	1883	237
scaffold13470	+	2315	9141	6827
scaffold7369	-	2973	5784	2812
scaffold21990	-	18	27051	27034
scaffold4400	+	19955	20316	362
scaffold4296	+	61565	61800	236
scaffold18493	+	2136	2716	581
scaffold16901	+	3868	4128	261
scaffold95056	-	155	391	237
scaffold7369	-	5423	6840	1418
scaffold66303	-	2018	2254	237
scaffold17486	-	2148	2384	237
scaffold87839	+	110	345	236
scaffold9840	-	642	1115	474
scaffold17486	-	928	1565	638
scaffold16674	-	152	831	680
scaffold4296	+	59710	60070	361
scaffold12450	+	10767	11101	335
scaffold37689	-	2188	2764	577
scaffold2587	-	4695	4930	236
scaffold2587	-	637	873	237
scaffold60729	+	144	380	237
scaffold58400	+	28	264	237
scaffold24254	+	4413	4648	236
scaffold22933	+	5865	6091	227
scaffold22618	+	120	356	237

scaffold85237	-	151	490	340
scaffold28766	-	9560	10028	469
scaffold22477	-	4053	4289	237
scaffold21653	-	4724	5382	659
scaffold1447	-	1832	2463	632
scaffold1447	-	667	904	238
scaffold547	-	3636	3872	237
scaffold34282	-	3783	4019	237
scaffold33079	-	3799	4035	237
scaffold33079	-	4513	4749	237
scaffold33079	-	4989	5225	237
scaffold33079	-	5227	5463	237
scaffold33079	-	3085	3321	237
scaffold33079	-	2609	2845	237
scaffold4544	+	2469	2705	237
scaffold23885	-	2644	3456	813
scaffold10662	-	8577	8831	255
scaffold4296	+	27595	27828	234
scaffold4296	+	25470	25703	234
scaffold16901	+	4715	5076	362
scaffold30453	-	96	332	237
scaffold19926	-	536	772	237
scaffold18931	-	3978	4214	237
scaffold10734	-	9980	10216	237
scaffold8568	+	12837	13198	362
scaffold4910	+	229	465	237
scaffold3525	+	11356	11592	237
scaffold17838	+	967	1203	237
scaffold942	-	316	678	363
scaffold8987	-	1066	2653	1588
scaffold2425	+	1323	1557	235
scaffold33591	-	1379	1615	237
scaffold19926	-	5162	5398	237
scaffold10662	-	3110	3346	237
scaffold9979	+	2366	2602	237
scaffold21237	-	239	696	458
scaffold5950	+	213	448	236
scaffold17956	+	5213	5426	214
scaffold9352	-	3828	4261	434
scaffold26766	-	1827	2063	237
scaffold19926	-	773	1124	352
scaffold30293	+	232	461	230
scaffold22804	-	3263	3499	237
scaffold10496	-	62	515	454
scaffold14078	+	294	880	587
scaffold22618	+	367	593	227
scaffold547	-	3385	8266	4882

scaffold10734	-	9113	9332	220
scaffold5950	+	4324	4685	362
scaffold37689	-	1825	2185	361
scaffold10734	-	14023	14236	214
scaffold16901	+	241	469	229
scaffold1447	-	1989	2210	222
scaffold1447	-	1737	1957	221
scaffold2735	+	749	1255	507
scaffold14773	+	13416	13644	229
scaffold19770	+	14339	14792	454
scaffold28620	-	1250	1447	198
scaffold9901	+	2	332	331
scaffold28620	-	89	284	196
scaffold9979	+	212	535	324
scaffold16986	+	3265	3480	216
scaffold3525	-	3641	3842	202
scaffold39889	+	8	205	198
scaffold18760	+	558	751	194
scaffold14769	-	115	315	201
scaffold13470	+	2788	3731	944
scaffold18533	-	7121	7304	184
scaffold8568	+	2665	2858	194
scaffold10734	-	2903	3083	181
scaffold18493	+	4940	5239	300
scaffold10734	-	6911	7096	186
scaffold16901	+	3684	3867	184
scaffold8676	-	8825	9243	419
scaffold8688	+	12278	12499	222
scaffold7168	-	5249	5645	397
scaffold8987	-	2765	3038	274
scaffold21990	-	26817	26973	157
scaffold35033	-	4268	4413	146
scaffold14773	+	292	1213	922
scaffold7168	-	3473	3621	149
scaffold7168	-	4250	4398	149
scaffold7168	-	4583	4731	149
scaffold7168	-	4694	4842	149
scaffold547	-	7793	8479	687
scaffold14773	+	162	291	130
scaffold13470	+	2277	3023	747
scaffold14773	+	7464	7585	122
scaffold37689	-	1692	1822	131
scaffold13470	+	3496	3972	477
scaffold17486	+	6677	6918	242
scaffold7436	+	10847	11550	704
scaffold17486	+	7065	7150	86
scaffold17486	+	6591	6676	86

scaffold13470	+	8907	8998	92
scaffold7436	+	11662	11752	91
scaffold17486	+	7862	7938	77
scaffold19485	+	494	583	90
scaffold19485	+	189	257	69
scaffold7369	-	3375	3406	32
scaffold195	+	5011	5044	34
scaffold7369	-	3176	3209	34
scaffold3525	-	7191	7218	28
scaffold35033	-	4201	4226	26
scaffold83421	-	1203	1226	24
scaffold13470	+	1223	1246	24
scaffold35033	-	4574	4606	33
scaffold3525	-	2079	2099	21

Table S3. The used primers and their parameters.

Primer Name	Sequence	Annealing, t °C	PCR Product Length, bp	References
CS237-f<br>CS237-1r	5′-GTGACGCTGGATGATTTCAA-3′<br>5′-GATGGGGGACACTCGTCTAA-3′	59.0	151	this article
CS237-f<br>CS237-2r	5′-GTGACGCTGGATGATTTCAA-3′<br>5′-GCCATCTTGACCGACTTCAT-3′	59.0	180	this article
CS237-1r<br>igs2004r	5′-GATGGGGGACACTCGTCTAA-3′<br>5′-GCAGGATCAACCAGGTAGCA-3′	50.0	-	this article, Hsieh et al. 2004 [42]
CS237-2r<br>igs2004r	5′-GCCATCTTGACCGACTTCAT-3′<br>5′-GCAGGATCAACCAGGTAGCA-3′	50.0	-	this article, Hsieh et al. 2004 [42]

Table S4. Levels of identity between the 126 bp and 111 bp subunits of the CS-237 consensus monomer, which were calculated using the GeneDoc software based on the alignment (see **Figure S2**).

Codes	126	111
**126**	100%	69%
**111**		100%

Table S5. Levels of identity between the delCS-237 monomers from the 52,978,632nd–52,979,130th bp region of the WRXK01000002 contig, which were calculated using the GeneDoc software based on the alignment (see **Figure 4**).

Codes	209	206	109
**209**	100%	89%	94%
**206**		100%	90%
**109**			100%

Table S6. Quantity and localization of HSPs for CS-237 and delCS-237 repeats based on BLAST alignment against the chromosome-level assembly of *C. sativa* (GCA_016165845.1).

CHR	Number of blastn HSPs with length > 200 and E-value < 1E-4		region	
	CS-237	delCS-237	CS-237	delCS-237
		any identity %:		
1	2	2	too few HSPs	
5	38	20	too few HSPs	
6	22865	10857	30M-35M	30M-35M
8	1919	999	0,4M-1,9M	0,4M-1,9M
9	2	2	too few HSPs	
X	132	65	54,65-54,67M	54,65-54,67M
		90+ identity %:		
6	3607	0	30M-35M	-
8	241	74	0,35M-0,75M	1,2M-1,95M
X	77	0	54,65-54,67M	-
